# Supplementary material for: Income distribution in Thailand is scale-invariant
Source: PLoS One. 2023 Jul 11;18(7):e0288265. doi: 10.1371/journal.pone.0288265 (PMC10335671; doi:10.1371/journal.pone.0288265)
Supplement: S1 Table — (PDF) [file pone.0288265.s001.pdf]

S1A Table. Income shares by quintile of Thailand from 1988 to 2021.

	1988	1990	1992	1994	1996	1998	2000	2002	2004	2006	2007	2009	2011	2013	2015	2017	2019	2021
**Q1**	0.0458	0.0429	0.0396	0.0407	0.0418	0.0430	0.0395	0.0418	0.0448	0.0379	0.0421	0.0442	0.0461	0.0416	0.0492	0.0502	0.0551	0.0550
**Q2**	0.0805	0.0754	0.0706	0.0735	0.0755	0.0775	0.0728	0.0769	0.0802	0.0763	0.0796	0.0826	0.0864	0.0900	0.0941	0.0915	0.0964	0.0971
**Q3**	0.1238	0.1170	0.1111	0.1167	0.1183	0.1200	0.1151	0.1206	0.1246	0.1217	0.1253	0.1265	0.1279	0.1351	0.1394	0.1373	0.1429	0.1418
**Q4**	0.2062	0.1950	0.1890	0.1968	0.1991	0.1982	0.1985	0.2011	0.2033	0.2016	0.2035	0.2028	0.1958	0.2082	0.2099	0.2101	0.2165	0.2138
**Q5**	0.5437	0.5697	0.5898	0.5723	0.5653	0.5613	0.5742	0.5595	0.5471	0.5625	0.5495	0.5439	0.5438	0.5251	0.5074	0.5109	0.4892	0.4923

Source: The Office of National Economic and Social Development Council. Poverty and income distribution statistics; 2023 [cited 2023 Feb. 15]. Available from: https://www.nesdc.go.th/main.php?filename=PageSocial

S1B Table. Income shares by decile of Thailand from 1988 to 2021.

	1988	1990	1992	1994	1996	1998	2000	2002	2004	2006	2007	2009	2011	2013	2015	2017	2019	2021
**D1**	0.0178	0.0169	0.0153	0.0159	0.0162	0.0168	0.0150	0.0161	0.0175	0.0134	0.0155	0.0162	0.0156	0.0106	0.0158	0.0182	0.0209	0.0204
**D2**	0.0280	0.0260	0.0242	0.0248	0.0256	0.0262	0.0245	0.0257	0.0273	0.0246	0.0266	0.0280	0.0305	0.0310	0.0334	0.0320	0.0342	0.0346
**D3**	0.0360	0.0335	0.0313	0.0325	0.0334	0.0344	0.0322	0.0340	0.0356	0.0334	0.0351	0.0366	0.0388	0.0404	0.0422	0.0410	0.0434	0.0439
**D4**	0.0445	0.0419	0.0393	0.0410	0.0421	0.0431	0.0406	0.0429	0.0446	0.0428	0.0445	0.0459	0.0476	0.0497	0.0519	0.0504	0.0530	0.0532
**D5**	0.0550	0.0519	0.0490	0.0517	0.0526	0.0533	0.0509	0.0535	0.0555	0.0539	0.0556	0.0565	0.0577	0.0610	0.0628	0.0618	0.0642	0.0642
**D6**	0.0689	0.0651	0.0620	0.0651	0.0657	0.0667	0.0642	0.0671	0.0690	0.0678	0.0697	0.0701	0.0702	0.0741	0.0766	0.0755	0.0787	0.0776
**D7**	0.0884	0.0832	0.0796	0.0839	0.0848	0.0848	0.0837	0.0859	0.0873	0.0867	0.0886	0.0884	0.0866	0.0916	0.0932	0.0929	0.0964	0.0953
**D8**	0.1178	0.1118	0.1095	0.1129	0.1142	0.1134	0.1148	0.1152	0.1161	0.1149	0.1149	0.1143	0.1092	0.1166	0.1167	0.1172	0.1201	0.1186
**D9**	0.1714	0.1654	0.1654	0.1650	0.1658	0.1662	0.1706	0.1648	0.1641	0.1626	0.1608	0.1595	0.1511	0.1577	0.1576	0.1581	0.1583	0.1580
**D10**	0.3723	0.4043	0.4244	0.4072	0.3995	0.3951	0.4036	0.3948	0.3830	0.3998	0.3887	0.3844	0.3927	0.3674	0.3498	0.3529	0.3308	0.3343

Source: The Office of National Economic and Social Development Council. Poverty and income distribution statistics; 2023 [cited 2023 Feb. 15]. Available from: https://www.nesdc.go.th/main.php?filename=PageSocial
