# Supplementary material for: Income distribution in Thailand is scale-invariant
Source: PLoS One. 2023 Jul 11;18(7):e0288265. doi: 10.1371/journal.pone.0288265 (PMC10335671; doi:10.1371/journal.pone.0288265)
Supplement: Attachment — Submitted filename: Response to Reviewers_R1.pdf [file pone.0288265.s002.pdf]

Response to Reviewer #1

Manuscript Number: PONE-D-23-12286R1

Title: Income distribution in Thailand is scale-invariant

I sincerely thank Reviewer #1 for providing several important comments and useful suggestions. I would like to inform Reviewer #1 that I have made a revision according to comments and suggestions made by Reviewer #1. I hope that my revised manuscript is clearer in all aspects that Reviewer #1 has commented and/or suggested. Let me respond to Reviewer #1's comments and suggestions as follows.

- Overall, I find the study interesting and worthy of investigation. However, I have several comments if addressed will improve the paper further and I outline them below.

I would like to thank Reviewer #1 for finding my paper interesting and worthy of investigation. I really appreciate it.

- Abstract. The abstract should be shortened. For publishing the paper, I suggest that author(s) deliver a concise and factual abstract. The abstract should state briefly the purpose of the research, the principal results and major conclusions. Readers are usually interested in highlighting ideas or arguments that are attractively presented in the abstract. For example, delete "…The concept of scale invariance or self-similarity is not well recognized by policymakers but may help explain the pattern of income distribution in Thailand that had not markedly changed over the past three decades...This requires creativity, openness to new ways of thinking, and disciplined analyses when it comes to policy design and implementation…".

I follow Reviewer #1's advice by deleting unnecessary sentences as suggested by Reviewer #1 above and only stating the purpose of the study, the principal results, major conclusions, and policy implications in Abstract in my revised manuscript.

- Although the authors make good attempt in providing a compelling case for the need of this study, but it can be strengthened further by spelling out more succinctly why the need to explore the nexus between the property of scale invariance and income distribution. This must come out clearly, as its present form, it has not been clearly articulated on.

In response to Reviewer #1's useful comment on this point, I explain the need to explore the link between scale invariance and income distribution in Introduction, paragraph 5 in my revised manuscript.

- The contribution section appears sketchy and general. I would like to see the contribution section strengthened drawing from the findings of the study.

I would like to thank Reviewer #1 for this very useful comment. I rewrote Discussion by first summarizing the findings and then trying to draw policy implications from the findings as stated in Discussion, paragraphs 1 and 2 in my revised manuscript. I also add suggestion for future research in Discussion, paragraph 4 in my revised manuscript.

- Related literature review with Thailand data is not clear. The author(s) should cite relevant related papers. For example, the study https://doi.org/10.1016/j.qref.2004.02.003 employ Thailand (and Asian) economic data.

In response to Reviewer #1 on this issue, I would like to clarify with Reviewer #1 that I obtained the data on income shares from the Office of National Economic and Social Development Council (NESDC) website which is cited in References in my revised manuscript. To make the data used in this study accessible and transparent, I also provide all data on income shares by quintile and by decile of Thailand from 1988 to 2021 in Supporting Information, S1 Table.

I would like to note that, although the NESDC website has an English language version, the income data can be accessed from its website in Thai language version only. The link to the NESDC website, which is https://www.nesdc.go.th/main.php?filename=PageSocial, is included in References in my revised manuscript. If readers go to this link and click "สถิติด้านความยากจนและการกระจายรายได้" which can be translated as "Poverty and income distribution statistics", readers can download the data on income shares by quintile and by decile of Thailand from 1988 to 2021 which are in Tables 8.2 and 8.5. The step-by-step of how to access the data is shown below.

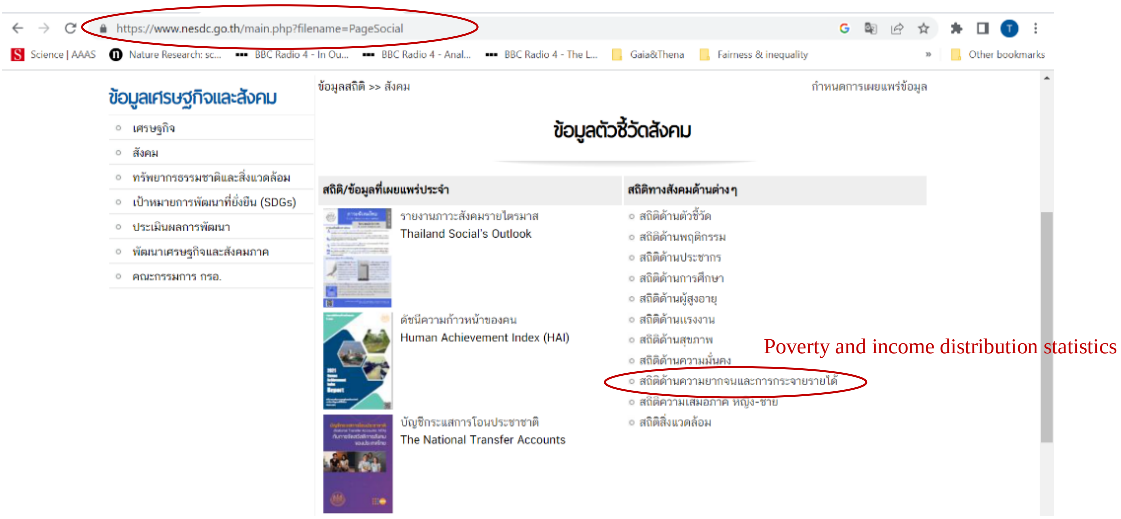

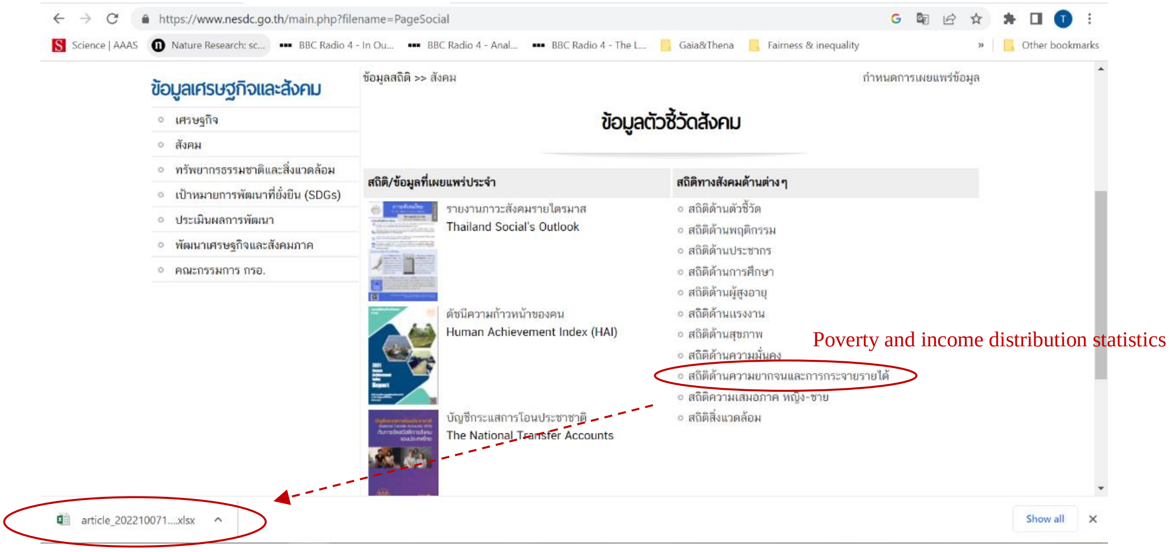

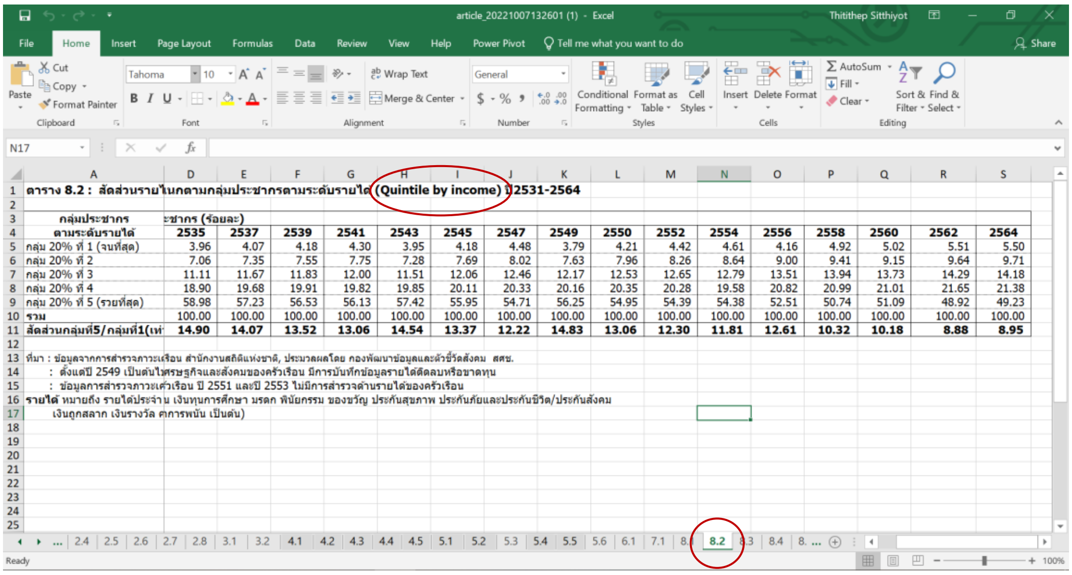

ตาราง 8.2 : สัดส่วนรายไนกตามกลุ่มประชากรตามระดับรายได้ (Quintile by income) ปี2531-2564

กลุ่มประชากร ตามระดับรายได้	2535	2537	2539	2541	2543	2545	2547	2549	2550	2552	2554	2556	2558	2560	2562	2564
กลุ่ม 20% ที่ 1 (จนที่สุด)	3.96	4.07	4.18	4.30	3.95	4.18	4.48	3.79	4.21	4.42	4.61	4.16	4.92	5.02	5.51	5.50
กลุ่ม 20% ที่ 2	7.06	7.35	7.55	7.75	7.28	7.69	8.02	7.63	7.96	8.26	8.64	9.00	9.41	9.15	9.64	9.71
กลุ่ม 20% ที่ 3	11.11	11.67	11.83	12.00	11.51	12.06	12.46	12.17	12.53	12.65	12.79	13.51	13.94	13.73	14.29	14.18
กลุ่ม 20% ที่ 4	18.90	19.68	19.91	19.82	19.85	20.11	20.33	20.16	20.35	20.28	19.58	20.82	20.99	21.01	21.65	21.38
กลุ่ม 20% ที่ 5 (รวยที่สุด)	58.98	57.23	56.53	56.13	57.42	55.95	54.71	56.25	54.95	54.39	54.38	52.51	50.74	51.09	48.92	49.23
รวม	100.00	100.00	100.00	100.00	100.00	100.00	100.00	100.00	100.00	100.00	100.00	100.00	100.00	100.00	100.00	100.00
**สัดส่วนกลุ่มที่5/กลุ่มที่1(เท่**	**14.90**	**14.07**	**13.52**	**13.06**	**14.54**	**13.37**	**12.22**	**14.83**	**13.06**	**12.30**	**11.81**	**12.61**	**10.32**	**10.18**	**8.88**	**8.95**

ที่มา : ข้อมูลจากการสำรวจภาวะเศรษฐกิจ สำนักงานสถิติแห่งชาติ, ประมวลผลโดย กองพัฒนาข้อมูลและตัวชี้วัดสังคม สศช.
: ตั้งแต่ปี 2549 เป็นต้นไปเศรษฐกิจและสังคมของครัวเรือน มีการบันทึกข้อมูลรายได้ติดลบหรือขาดทุน
: ข้อมูลการสำรวจภาวะเศรษฐกิจครัวเรือน ปี 2551 และปี 2553 ไม่มีการสำรวจด้านรายได้ของครัวเรือน
รายได้ หมายถึง รายได้ประจำ เงินทุนการศึกษา มรดก พินัยกรรม ของขวัญ ประกันสุขภาพ ประกันภัยและประกันชีวิต/ประกันสังคม
เงินถูกสลาก เงินรางวัล การพนัน เป็นต้น)

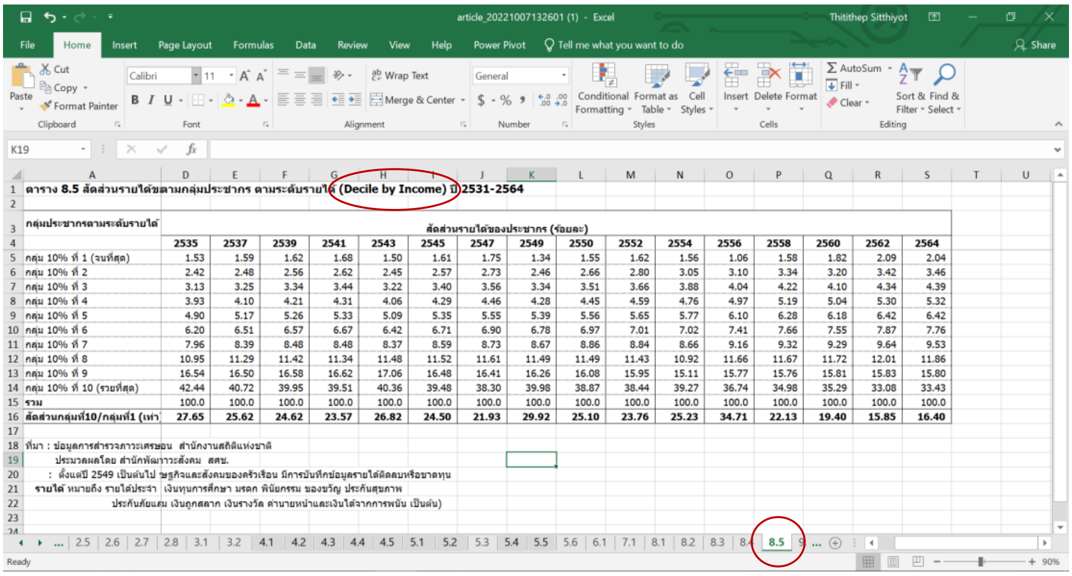

ตาราง 8.5 สัดส่วนรายได้ของตามกลุ่มประชากร ตามระดับรายได้ (Decile by Income) ปี 2531-2564

กลุ่มประชากรตามระดับรายได้	สัดส่วนรายได้ของประชากร (ร้อยละ)															
	2535	2537	2539	2541	2543	2545	2547	2549	2550	2552	2554	2556	2558	2560	2562	2564
กลุ่ม 10% ที่ 1 (จนที่สุด)	1.53	1.59	1.62	1.68	1.50	1.61	1.75	1.34	1.55	1.62	1.56	1.06	1.58	1.82	2.09	2.04
กลุ่ม 10% ที่ 2	2.42	2.48	2.56	2.62	2.45	2.57	2.73	2.46	2.66	2.80	3.05	3.10	3.34	3.20	3.42	3.46
กลุ่ม 10% ที่ 3	3.13	3.25	3.34	3.44	3.22	3.40	3.56	3.34	3.51	3.66	3.88	4.04	4.22	4.10	4.34	4.39
กลุ่ม 10% ที่ 4	3.93	4.10	4.21	4.31	4.06	4.29	4.46	4.28	4.45	4.59	4.76	4.97	5.19	5.04	5.30	5.32
กลุ่ม 10% ที่ 5	4.90	5.17	5.26	5.33	5.09	5.35	5.55	5.39	5.56	5.65	5.77	6.10	6.28	6.18	6.42	6.42
กลุ่ม 10% ที่ 6	6.20	6.51	6.57	6.67	6.42	6.71	6.90	6.78	6.97	7.01	7.02	7.41	7.66	7.55	7.87	7.76
กลุ่ม 10% ที่ 7	7.96	8.39	8.48	8.48	8.37	8.59	8.73	8.67	8.86	8.84	8.66	9.16	9.32	9.29	9.64	9.53
กลุ่ม 10% ที่ 8	10.95	11.29	11.42	11.34	11.48	11.52	11.61	11.49	11.49	11.43	10.92	11.66	11.67	11.72	12.01	11.86
กลุ่ม 10% ที่ 9	16.54	16.50	16.58	16.62	17.06	16.48	16.41	16.26	16.08	15.95	15.11	15.77	15.76	15.81	15.83	15.80
กลุ่ม 10% ที่ 10 (รวยที่สุด)	42.44	40.72	39.95	39.51	40.36	39.48	38.30	39.98	38.87	38.44	39.27	36.74	34.98	35.29	33.08	33.43
รวม	100.0	100.0	100.0	100.0	100.0	100.0	100.0	100.0	100.0	100.0	100.0	100.0	100.0	100.0	100.0	100.0
**สัดส่วนกลุ่มที่10/กลุ่มที่1 (เท่า)**	**27.65**	**25.62**	**24.62**	**23.57**	**26.82**	**24.50**	**21.93**	**29.92**	**25.10**	**23.76**	**25.23**	**34.71**	**22.13**	**19.40**	**15.85**	**16.40**

ที่มา : ข้อมูลการสำรวจภาวะเศรษฐกิจ สำนักงานสถิติแห่งชาติ
ประมวลผลโดย สำนักพัฒนาภาวะสังคม สศช.
: ตั้งแต่ปี 2549 เป็นต้นไป ษฐกิจและสังคมของครัวเรือน มีการบันทึกข้อมูลรายได้ติดลบหรือขาดทุน
รายได้ หมายถึง รายได้ประจำ เงินทุนการศึกษา มรดก พินัยกรรม ของขวัญ ประกันสุขภาพ
ประกันภัยแถม เงินถูกสลาก เงินรางวัล ค่านายหน้าและเงินได้จากการพนัน เป็นต้น)

- It is not properly motivated the use of the pairwise K-S test.

In response to Reviewer #1's comment on the use of pairwise K-S test, in Introduction, paragraph 4 in my revised manuscript, besides the study by Chakrabarti et al. (2013) who note that there tends to be an agreement among academic scholars in economics, statistics, and physics that the upper tail of income distribution could be well described by power law but the lower part of income distribution could be fitted by power law, gamma, or log-normal distribution, I include additional studies, namely, Montroll and Shlesinger (1982), Champernowne and Cowell (1998), Drăgulescu and Yakovenko (2001), Chatterjee and Chakrabarti (2007), and Yakovenko and Barkley Rosser (2009), all of which use complicated statistical distributions in order to fit the income data and test the property of scale invariance as a ground for using the pairwise K-S test which is relatively simpler and does not require a priori assumption with regard to the distribution of data but could achieve the same task.

In addition, in Materials and Methods, paragraph 2, I explain the reason for using the K-S test by referring to the study by Sitthiyot et al. (2020) who note that the K-S test is non-parametric and commonly used to determine if two datasets differ statistically. Its main advantages are that it makes no assumption about the distribution of data and works for small sample size.
